# Paclitaxel Restores Sensitivity to Chemotherapy in Preclinical Models of Multidrug-Resistant Intrahepatic Cholangiocarcinoma

**DOI:** 10.3389/fonc.2022.771418

**Published:** 2022-02-17

**Authors:** Annamaria Massa, Caterina Peraldo-Neia, Francesca Vita, Chiara Varamo, Marco Basiricò, Chiara Raggi, Paola Bernabei, Jessica Erriquez, Ivana Sarotto, Francesco Leone, Serena Marchiò, Giuliana Cavalloni, Massimo Aglietta

**Affiliations:** ^1^ Medical Oncology, Candiolo Cancer Institute, Fondazione del Piemonte per l'Oncologia (FPO)-Istituto di Ricovero e Cura a Carattere Scientifico (IRCCS), Torino, Italy; ^2^ Division of Oncology, University of Torino, Torino, Italy; ^3^ Laboratory of Cancer Genomics, Fondazione Edo ed Elvo Tempia, Biella, Italy; ^4^ Department of Experimental and Clinical Medicine, University of Firenze, Firenze, Italy; ^5^ Flow Cytometry Center, Candiolo Cancer Institute, Fondazione del Piemonte per l'Oncologia (FPO)-Istituto di Ricovero e Cura a Carattere Scientifico (IRCCS), Torino, Italy; ^6^ Candiolo Cancer Institute, Fondazione del Piemonte per l'Oncologia (FPO)-Istituto di Ricovero e Cura a Carattere Scientifico (IRCCS), Torino, Italy; ^7^ Unit of Pathology, Candiolo Cancer Institute, Fondazione del Piemonte per l'Oncologia (FPO)-Istituto di Ricovero e Cura a Carattere Scientifico (IRCCS), Torino, Italy; ^8^ Department of Oncology, Azienda Sanitaria Locale (ASL) Biella (BI), Nuovo Ospedale degli Infermi, Biella, Italy

**Keywords:** biliary tract cancers, intrahepatic cholangiocarcinoma (iCCA), gemcitabine resistance, paclitaxel, apoptosis

## Abstract

The treatment of unresectable cholangiocarcinoma (CCA) is limited by the development of resistance to conventional first-line chemotherapy based on gemcitabine (GEM). In addition, a prior treatment with GEM frequently induces cross-resistance to other drugs employed in the second-line. Paclitaxel (PTX) is now emerging as an alternative option for the management of advanced/metastatic CCA. In the present work, we evaluate the antitumor activity of PTX in preclinical models of multidrug-resistant intrahepatic cholangiocarcinoma (iCCA). *In vitro*, PTX decreases tumor cell viability by affecting the cell cycle and inducing apoptosis and impairs the stem cell compartment. *In vivo*, a therapeutic regimen containing albumin-bound nanoparticle (Nab)-PTX overcomes drug resistance resulting in delayed tumor growth, impaired organization of the tumor vasculature, and reduced glucose uptake. Together, our results provide a rationale to consider PTX-based regimens in patients with iCCA who became refractory to conventional therapies.

## Introduction

Cholangiocarcinomas (CCAs) are heterogeneous biliary epithelial tumors arising from the intrahepatic (iCCA), perihilar (pCCA), or distal biliary (dCCA) tree epithelium (1). CCA is the second most common hepatic malignancy after hepatocellular carcinoma (HCC), and the overall incidence has progressively increased worldwide over the past four decades (1). Given the aggressive course and the lack of early symptoms, diagnosis often occurs at an advanced stage, resulting in poor prognosis and in a 5-year survival rate of ~10% (2). Even when the disease is amenable to surgery, the reported 5-year survival remains low, ranging from 20% to 50% (3). The unavailability of molecular markers for early diagnosis, the limited therapeutic options, and the absence of effective targeted therapies remain the major clinical hurdles in CCA management.

Randomized clinical trials in the advanced disease setting have established gemcitabine (GEM) monotherapy or GEM–platinum combination as first-line chemotherapies (3–5). The effectiveness of GEM, however, is limited by the frequent development of resistance (6). The mechanisms, mostly investigated in pancreatic cancer, include the downregulation of the GEM cellular transporters, the alteration of proteins related to GEM catabolism, and the activation of alternative pathways in DNA repair and in resistance to apoptosis, including the promotion of epithelial-to-mesenchymal transition (EMT) (7). Upon progression, a substantial proportion of CCA patients are redirected to a fluoropyrimidine (FP)-based regimen, namely, 5-fluorouracil (5-FU) or its prodrug capecitabine (8, 9). Despite the broadly accepted clinical indication, a systematic review of 25 studies retrieved insufficient evidence in support of this second-line therapy (10). Accordingly, in a retrospective analysis on 321 patients previously treated with first-line GEM–cisplatin, the overall response rate (ORR) to second-line FP-based chemotherapy was as low as 3%, the median progression-free survival (PFS) 1.9 months only, and the median overall survival (OS) 6.5 months (11). Similar modest results are reported in the successive studies by Takahara et al. (139 patients, ORR 9%, PFS 2.8, OS 7.7) (12) and Schweitzer et al. (144 patients, ORR 9.7%, OS 9.9) (8). In recent work, we demonstrated that both primary and acquired GEM resistance confer cross-resistance to other chemotherapeutics, including 5-FU in addition to carboplatin and trabectedin (13, 14), which could explain the disappointing outcome of conventional second-line therapy in CCA patients.

An emerging drug for CCA treatment, paclitaxel (PTX) is a taxane that stabilizes cell microtubules, thus causing an arrest of the cell cycle (15, 16). Due to its strong cytotoxic action, PTX entered the therapeutic armamentarium of a variety of malignancies, including neoplasms of gastrointestinal origin (17, 18). Low-dose PTX (below the threshold of toxicity) is sufficient to downregulate S100A4 protein expression in CCA cell lines, with consequent decreased cell motility and invasiveness *in vitro* and reduced hematogenous dissemination *in vivo* (6). TGF-β1-driven EMT is also inhibited (19). Preliminary studies reported the clinical efficacy of a nanocarrier-based formulation of PTX (albumin-bound nanoparticle, Nab-PTX), in combination with GEM, as first-line treatment for advanced/metastatic CCA patients (20, 21). Other nanocarrier derivatives of PTX have also been investigated in combination with GEM in cell models (22) and in a Phase II clinical trial as first-line chemotherapy for unresectable/metastatic CCA (23), with encouraging results.

In a small Phase I trial, the metronomic administration of PTX as third-line palliative chemotherapy in patients with advanced biliary tract cancers was well-tolerated, with clinical response in 5 out of 6 cases (24). More recently, Nab-PTX has been evaluated in a second-line pilot study in combination with capecitabine in CCA patients who progressed after GEM–platinum combination. In this trial, a disease control rate of 80% (8 out of 10 patients) and a median PFS and OS of 5.7 and 12.1 months, respectively, were achieved (25). Together, these results support the inclusion of Nab-PTX in therapeutic regimens for patients who have developed resistance to GEM.

The limits of current CCA management highlight the need of therapeutic options that could overcome GEM resistance. In a previous study of drug prediction analysis, we identified PTX as a therapeutic opportunity in a model of GEM-resistant iCCA (14), while—to the best of our knowledge—there are no data on the outcome of PTX treatment in CCA with multidrug resistance. To fill this gap, we here investigated the efficacy of PTX (*in vitro*) and Nab-PTX (*in vivo*), either alone or in combination with GEM, in preclinical models of iCCA with multidrug resistance.

## Materials and Methods

### Cell Lines

The multidrug-resistant iCCA cell lines MT-CHC01R1.5 (with acquired resistance to GEM) and 82.3 (with primary resistance to GEM), isolated and stabilized at our Institution (13, 14, 26), were maintained in KnockOut DMEM/F-12 (Thermo Fisher Scientific, Waltham, MA, USA) supplemented with 10% fetal bovine serum (FBS), Hepes buffer, 100 U/ml penicillin, and 100 μg/ml streptomycin (P/S) (all from Sigma-Aldrich, St. Louis, MO, USA).

### Drugs

GEM hydrochloride (Sandoz, Novartis Division, Siena, Italy) and PTX (Sigma-Aldrich) were dissolved in water for injection and aliquoted in working solutions. For the *in vivo* experiments, Nab-PTX (Abraxane, Celgene, Milan, Italy) was used as PTX formulation.

### Cell Growth Assay

Cell viability was evaluated by plating 2,500 cells per well into 96-well plates in optimal culture medium. After 24 h, cells were treated with escalating doses of PTX (from 0.9375 to 15 ng/ml). Viability was quantified after 72 h with the Cell Titer-Glo^®^ commercial kit (Promega, Milan, Italy), and luminescence was quantified with a GloMax microplate reader (GloMax-Multi Detection System, Promega). The IC50 of the drug was calculated using the CalcuSyn software (Biosoft, Cambridge, UK) based on the Chou-Talalay method.

### Cell Cycle and Apoptosis Analysis

For the cell cycle analysis, 2 × 10^6^ cells were plated into 10-cm plates. After 24 h, cells were treated with GEM (1.5 µM), PTX (15 ng/ml), or their combination; after 48 h of treatment, cells were fixed with ice-cold 70% ethanol. Cells were then incubated at 37°C for 15 min in the dark in PBS containing 10 μg/ml of propidium iodide (PI) (Thermo Fisher Scientific), 0.1% (v/v) Triton X-100, and 100 μg/ml RNase. Samples were then acquired with a CyAn ADP™ flow cytometer (Beckman Coulter, Brea, CA, USA) and data analyzed with the Summit software v4.3 (Beckman Coulter). The percentage of cells in the various phases of the cell cycle (G0/G1, S, G2/M) was calculated. Three independent experiments were performed.

For the apoptosis analysis, 1 × 10^5^ cells were seeded per well of 6-well plates. Cells were treated after 24 h with GEM (1.5 µM), PTX (15 ng/ml), or their combination. The quantification of apoptotic cells was performed after 48 h with the Annexin V-allophycocyanin (APC)/PI staining assay. Briefly, supernatants were recovered and cells were preincubated for 30 min at 4°C in the dark with a commercial binding buffer (Bender MedSystems, Wien, Austria). Next, cells were suspended in binding buffer containing Annexin V-APC (Bender MedSystems) and PI 50 µg/ml (Thermo Fisher Scientific) and incubated for 1 h at 4°C in the dark. Samples (30,000 events/experimental point) were acquired with a CyAn ADP™ flow cytometer (Beckman Coulter). Cell death was expressed as the percent average of dead cells (early and late apoptotic) obtained in three independent experiments.

### Western Blot

Cells were subjected to lysis with lysis buffer (Cell Signaling, Danvers, MA, USA) and centrifuged at 20,000x*g* for 30 min at 4°C. Twenty μg of proteins was electrophoresed on Mini-PROTEAN TGX Precast Gels (4%–20%) and transferred on nitrocellulose Midi membranes using Trans-Blot Turbo (Bio-Rad, Hercules, CA, USA). Non-specific sites were blocked with 5% non-fat dry milk (Bio-Rad Laboratories, Munchen, Germany), and then the blots were stained using standard procedures with Cleaved Caspase 3 (CC3) and cleaved poly(ADP-ribose) polymerase (cPARP) primary (Cell Signaling) antibodies. Finally, signals were revealed by a chemiluminescence reagent (Euroclone, Milan, Italy). Densitometric analysis was performed with Fiji ImageJ (27) using the Analyze/Gel function. Briefly, after selecting each lane with a rectangular selector, densitometric histograms were derived with the Plot Lanes function and the peaks were measured excluding the side curves that originated from background signals.

### Cholangiosphere Formation Assay

For the sphere formation assay, 2.5 × 10^5^ cells were seeded per well into 24-well ultra-low attachment plates in serum-free DMEM-F12 (Sigma) with 1× B27 Supplement (Thermo Fisher Scientific), 200 ng/ml human EGF (PeproTech, London, UK), 0.4% bovine serum albumin (BSA) (Sigma), 4 μg/ml insulin (Sigma), and P/S (Life Technologies). Cells were treated with GEM (1.5 µM) or PTX (15 ng/ml). After 7 days, spheres with a diameter >50 µm were counted under an optical microscope (DMIL, Leica Microsystems GmbH). Three fields per well were acquired, and three independent experiments were performed.

### *In Vivo* Antitumor Activity of PTX in Multidrug-Resistant iCCA Models

NOD-SCID (Non-Obese Diabetic-Severe Combined Immunodeficient) female mice (4–6 weeks old) (Charles River Laboratory, Wilmington, MA, USA) were maintained under sterile conditions in micro-isolator cages at the animal facilities of Candiolo Cancer Institute, FPO-IRCCS. All animal procedures were approved by the Institutional Ethical Committee for Animal Experimentation (Fondazione Piemontese per la Ricerca sul Cancro) and by the Italian Ministry of Health (Ethic code: 177/2015-PR; 178/2015-PR and 106/2021-PR). In three independent experiments, MT-CHC01R1.5 cells resuspended in 50% (v/v) growth factor-reduced basement membrane matrix (Matrigel, BD Bioscience) were injected subcutaneously into the right flank of mice. When tumors reached a volume of 80–100 mm^3^ (approximately 3 weeks after injection), animals were randomized into four arms of treatment (6 mice/arm) that were treated twice a week with vehicle only (NT), GEM (25 mg/kg), Nab-PTX (10 mg/kg), or their combination. Tumor growth was measured with a caliper twice a week until the end of the experiment. Tumor volumes were calculated using the formula *V* = (*A* × *B* ^2^)/2 (*V* = tumor volume, *A* = largest diameter, *B* = smallest diameter). In addition, at the end of the experiments, tumors were explanted and weighted. Glucose uptake was analyzed in live animals using IVIS Lumina II for 2D acquisitions and IVIS Spectrum CT for 3D acquisition (Perkin Elmer Waltham, MA, USA) after tail vein injection of XenoLightRediJect 2-DeoxyGlucosone (DG)-750 (Caliper Life Sciences, Waltham, MA, USA). Images were analyzed with the Living Image Software (PerkinElmer, Caliper Life Sciences). To calculate glucose uptake by tumor xenografts, the ratio between total fluorescence emitted and total number of tumor mass voxels was calculated.

### Histochemistry and Immunohistochemistry

To evaluate the presence of necrosis, tumors were formalin-fixed and paraffin-embedded (FFPE) and 3-μm-thick sections were stained with hematoxylin and eosin (H&E). The Polygon Selection tool of ImageJ software was used to measure necroti areas. Five fields of each slide were analyzed. The proliferation marker Ki67, the apoptosis marker CC3, and the endothelial cell marker CD146 were investigated by immunohistochemistry (IHC). Briefly, 5-μm FFPE tissue sections were deparaffinized, rehydrated, and permeabilized, followed by antigen retrieval and saturation. Next, sections were decorated with the following primary antibodies: anti-Ki67 (DAKO, Carpinteria, CA, USA), anti-CC3 (Cell Signaling), and anti-CD146 (Abcam, Cambridge, UK). HRP-conjugated secondary antibodies (Anti-Mouse or Anti-Rabbit, DAKO) were applied, and the signal was detected with a diaminobenzidine tetrahydrochloride (DAB)-based system (Liquid DAB chromogen system, DAKO). Slides were counterstained with hematoxylin. Proliferation rates were calculated as the percentage of Ki67-positive cells out of the total number of cells in five fields of each slide. Apoptosis was expressed as the absolute value of CC3-positive cells in the total area of each slide. Tumor vasculature was evaluated by CD146 immunostaining; the result was represented as percentage of CD146-positive area in five fields of each slide. For this purpose, the Color Deconvolution plugin (ImageJ) was used.

### Statistical Analysis

Experiments were analyzed using mean ± standard error of mean (SEM) or standard deviation (SD), as indicated in the figure legends. Two-way ANOVA was used to analyze cell and tumor growth, as well as the differences in terms of drug response. One-way ANOVA (multiple comparisons) was used for the other statistical analysis. *p < 0.05; **p < 0.01; ***p < 0.001; and ****p < 0.0001 were considered statistically significant.

## Results

### PTX Inhibits Viability, Perturbs the Cell Cycle, and Induces Apoptosis in Multidrug-Resistant iCCA Cell Lines

**Figure 1:**
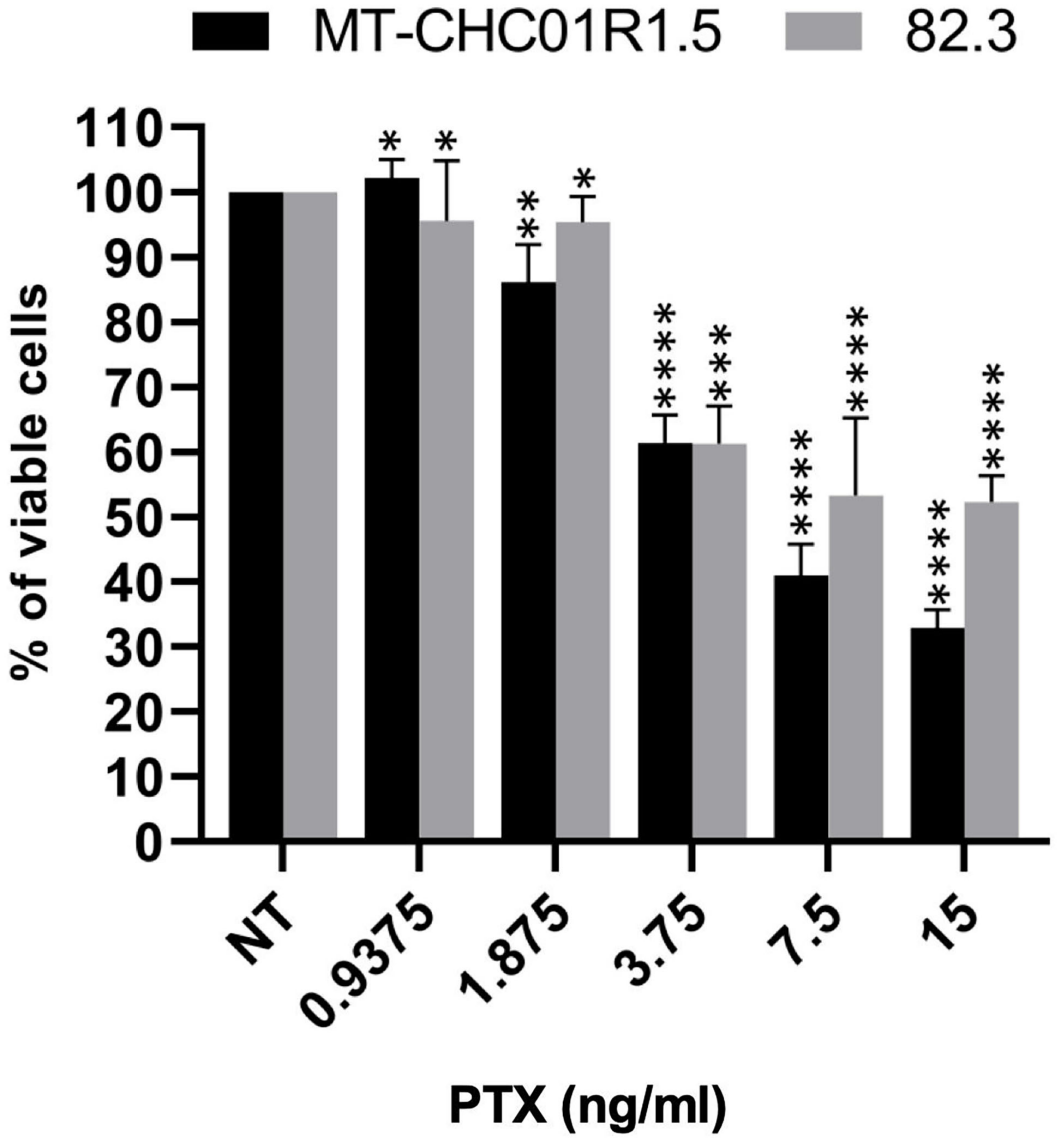
Cytotoxicity of PTX in multidrug-resistant iCCA cell lines. Representative histograms showing the effect of PTX on cell viability in MT-CHC01R1.5 and 82.3 cells treated for 72 h with the indicated drug concentrations. IC50 values were calculated from three independent experiments performed in quadruplicate. Mean ± SEM of each experimental point are represented *p < 0.05; **p < 0.01; ***p < 0.001; and ****p < 0.0001.

We first quantified the cytotoxic effect of PTX on two multidrug-resistant iCCA cell lines, namely, MT-CHC01R1.5 (acquired resistance to GEM) and 82.3 (primary resistance to GEM) (13, 14). Cells were incubated for 72 h with escalating doses of the drug, followed by a cell viability assay (**Figure 1**). The derived IC50 values were calculated as 4.81 ± 1.98 and 10.55 ± 0.77 ng/ml for MT-CHC01R1.5 and 82.3 cells, respectively.

**Figure 2:**
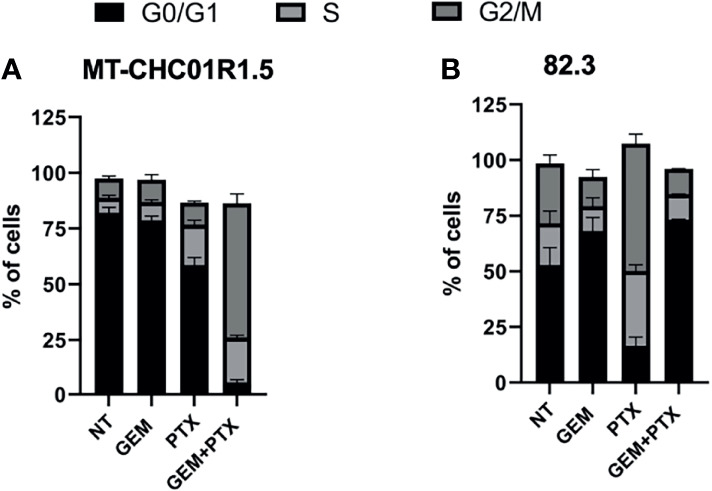
Cell cycle perturbation by PTX in multidrug-resistant iCCA cell lines. Cell cycle analysis of MT-CHC01R1.5 **(A)** and 82.3 **(B)** cells treated for 48 h in the absence (NT) and in the presence of the following drugs: GEM (1.5 µM), PTX (15 ng/ml), or GEM + PTX. The histograms represent the percent mean ± SD of cells in the various phases of the cell cycle (G0/G1, S, G2/M) in three independent experiments.

We next analyzed the cell cycle after treatment with GEM (1.5 μM), PTX (15 ng/ml), or GEM (1.5 μM) + PTX (15 ng/ml) for 48 h. As shown in **Figure 2** and **Supplementary Table 1** in both cell lines, as expected due to the previously characterized resistance, treatment with GEM did not induce any change in the cell cycle and was therefore considered as an additional negative control. In MT-CHC01R1.5 cells, treatment with PTX induced a decrease of cells in the G0/G1 phase compared to both NT and GEM and an increase of cells in the S phase compared to both NT and GEM. Treatment with GEM + PTX induced a drastic decrease of cells in the G0/G1 phase compared to all other experimental points, an increase of cells in the S phase compared to both NT and GEM, and a massive increase of cells in the G2/M phase compared to all other experimental points. In 82.3 cells, treatment with PTX induced a substantial decrease of cells in the G0/G1 phase compared to both NT and GEM, an increase of cells in the S phase compared to both NT and GEM, and a considerable increase of cells in the G2/M phase compared to both NT and GEM. Treatment with GEM + PTX was not different from GEM alone.

**Figure 3:**
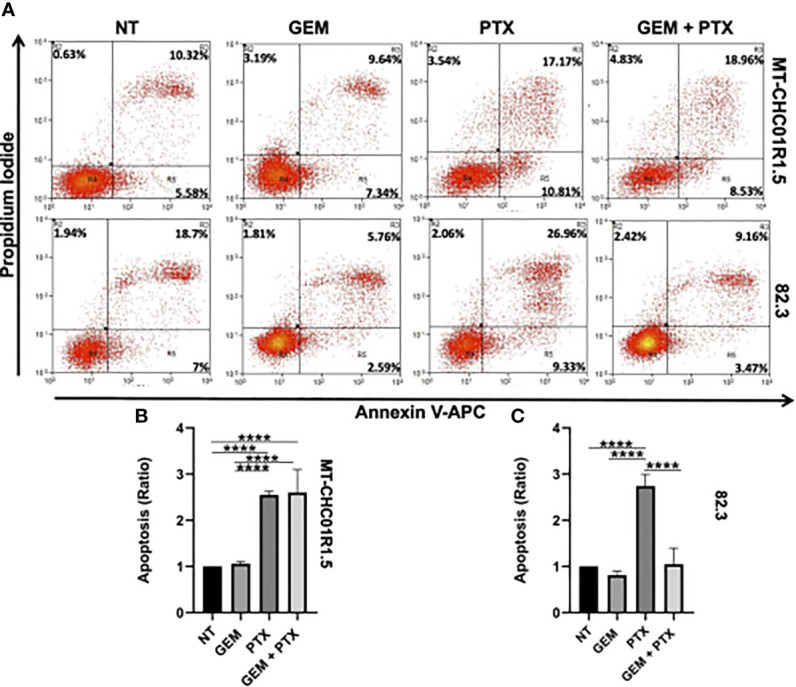
Pro-apoptotic activity of PTX in multidrug resistant iCCA cell lines. Representative dot plots **(A)** and histograms of cell death flow cytometry analysis on MT-CHC01R1.5 **(B)** and 82.3 **(C)** cell lines, untreated and treated with GEM (1.5 µM), PTX (15 ng/mL), or GEM + PTX. The percent average of apoptotic cells (early and late apoptosis) was analyzed, and the ratio between treatments and NT was calculated. The histograms represent the mean ± SEM of values obtained in three independent experiments ****p <0.0001.

We further characterized drug efficacy in terms of cell death induced by 48 h of incubation with GEM (1.5 μM), PTX (15 ng/ml), or GEM (1.5 μM) + PTX (15 ng/ml). After treatment, cells were stained with APC-labeled Annexin V (a marker of early apoptosis) and PI (a marker of late apoptosis and necrosis), followed by flow cytometry. Early (positivity to Annexin V) and late apoptosis (positivity to both PI and Annexin V) were induced by PTX alone in both cell lines. The addition of gemcitabine did not increase the apoptotic fraction on MT-CH01R1.5 cells, while in 82.3, GEM acts as a PTX antagonist (**Figure 3**).

A Western blot analysis of two apoptotic markers, namely, CC3 and cPARP confirmed the dissimilar behavior of the two cell models (**Figure S1**). In the case of MT-CH01R1.5 cells, this analysis fully mirrored the flow cytometry results: both CC3 and cPARP were upregulated by PTX alone or in combination. For 82.3 cells, the results were discordant: CC3 was downregulated by GEM-containing treatments, while cPARP was upregulated by PTX-containing treatments (necrosis, positivity to PI) was slightly induced by PTX alone or in combination in MT-CHC01R1.5 cells but not in 82.3 cells.

Together, these data suggest that PTX *in vitro*, alone, exerted a cytotoxic effect in both cell lines. In 82.3 cells, the cytostatic effect of GEM prevailed in the drug combination, resulting as an antagonist of PTX.

### PTX Reduces the Putative Stem Cell Compartment in Multidrug-Resistant iCCA Cell Lines

**Figure 4:**
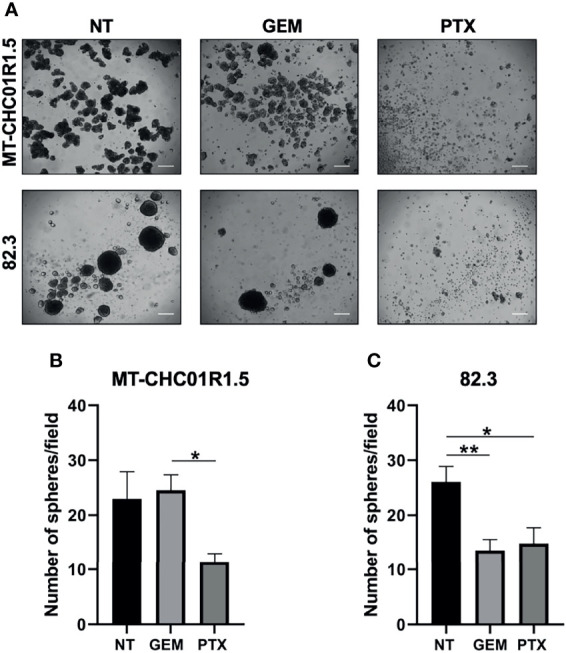
Inhibitory effect of PTX on the cancer stem cell compartment of multidrug-resistant iCCA cell lines. **(A)** Representative images of cholangiospheres obtained from MT-CHC01R1.5 and 82.3 cells untreated (NT) and treated with GEM (1.5 µM), PTX (15 ng/ml). Images were acquired with an optical microscope at ×4 magnification; scale bar, 50 µm. **(B, C)** Quantification of cholangiospheres after 7 days of treatment. Spheres with a diameter >50 µm were counted, and the average of 5 fields per image of three independent experiments was calculated. Histograms represent the mean ± SEM of these values. *p < 0.05; **p < 0.01.

We tested the ability of cells to grow in a non-adherent, serum-free environment, an assay commonly used to quantify cancer cell subpopulations with stemness properties, which are usually more refractory to chemotherapy (28, 29). The effect of PTX on the stem cell compartment was assessed on MT-CHC01R1.5 and 82.3 cells as their capacity to form spheroids (cholangiospheres) when grown in suspension and in the presence of GEM (1.5 μM) or PTX (15 ng/ml). Cholangiospheres were imaged and quantified under a light microscope after 7 days of treatment (**Figure 4A**). In 82.3 cells, but not in MT-CHC01R1.5, GEM was capable of reducing the numbers of presumed cancer stem cells. In both cell lines, PTX reduced the number of cholangiospheres compared to the controls NT or GEM (**Figures 4B, C**).

These results suggest that PTX may overcome drug resistance by affecting a subpopulation of cells with stemness features.

### A Combination of Nab-PTX and GEM Delays the Growth of Multidrug-Resistant iCCA Xenografts

**Figure 5:**
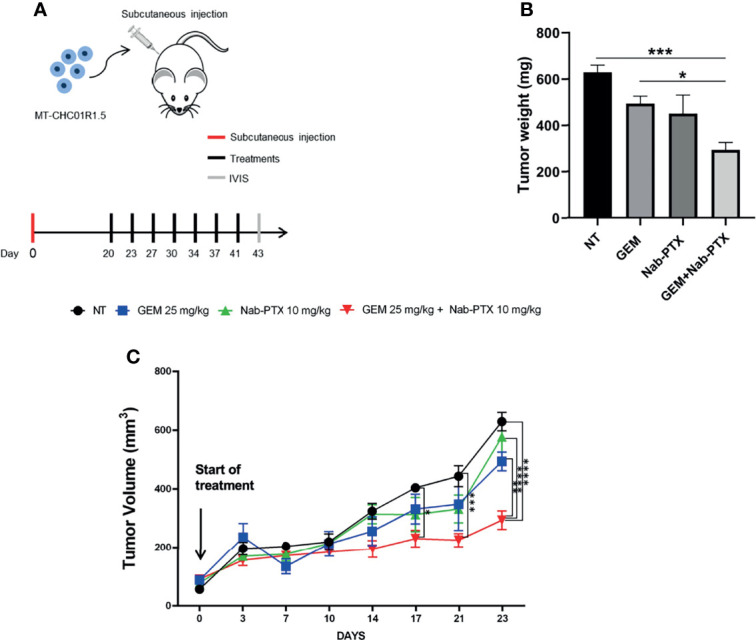
*In vivo* antitumor activity of GEM + Nab-PTX in MT-CHC01R1.5 xenografts. **(A)** Schematic representation of the experimental plan: red line, day of subcutaneous injection of MT-CHC01R1.5 in NOD/SCID mice; black lines, days of treatment; gray line, imaging and sacrifice. Treatments: NT (vehicle), GEM (25 mg/kg), Nab-PTX (10 mg/kg), GEM (25 mg/kg) + Nab-PTX (10 mg/kg). **(B)** Weight of the explanted tumor masses at the end of the experiment. The histogram represents mean ± SEM of values in each treatment arm. **(C)** Growth curve of MT-CHC01R1.5 xenografts throughout the 23 days of treatment. Values represent mean ± SEM of tumor volumes in each treatment arm at the indicated timepoints *p < 0.05; **p < 0.01; ***p < 0.001; and ****p < 0.0001.

**Figure 6:**
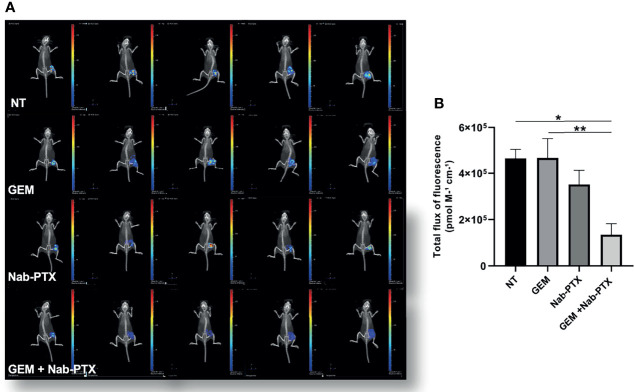
Inhibitory effect of GEM + Nab-PTX on glucose uptake. **(A)** Visualization of tumor-glucose uptake following injection of the fluorescent glucose derivative 2 DG 750 and imaging with an IVIS instrument. Treatments: NT (vehicle), GEM (25 mg/kg), Nab-PTX (10 mg/kg), GEM (25 mg/kg) + Nab-PTX (10 mg/kg). **(B)** Cumulative analysis of total flux of fluorescence uptaken by the tumor masses. The histogram represents the mean ± SEM of the values *p < 0.05; **p < 0.01.

Having demonstrated the feasibility of PTX-based preclinical applications in multidrug-resistant iCCA cell lines, we set up the corresponding *in vivo* experiment. Following a recent study that demonstrated the efficacy of Nab-PTX over PTX in animal models (30), we used Nab-PTX, either as a single agent or in combination with GEM, for our *in vivo* studies with MT-CHC01R1.5 xenografts. Three weeks after injection, when tumor volumes reached 80–100 mm^3^, mice were randomized into four arms of treatment: (i) drug vehicle (saline solution) administered intraperitoneally (i.p.) (NT), (ii) GEM (i.p., 25 mg/kg), (iii) Nab-PTX (i.p., 10 mg/kg), and (iv) drug combination. GEM and Nab-PTX were administered every 3 days (**Figure 5A**). Tumors were monitored and measured twice a week. After a total of 23 days of treatments, animals were euthanized and tumor masses explanted (**Supplementary Figure 1**). **Figures 5B, C** show that the combination of GEM and Nab-PTX delayed tumor growth, in terms of both tumor weight, which was reduced in the drug combination arm compared with NT (p < 0.001) and single-agent GEM (p < 0.05) (**Figure 5B**), and tumor volume, lower in the drug combination arm compared with NT (p < 0.0001), Nab-PTX (p < 0.0001), and GEM (p < 0.01) (**Figure 5C**). The response in terms of tumor growth was also evaluated by 3D (*in vivo*) and 2D (*ex vivo*) imaging upon injection of the fluorescent glucose derivative 2GD 750. In live animals, the combination of GEM and Nab-PTX reduced glucose uptake compared to NT (p < 0.05) and GEM (p < 0.01), as shown in **Figure 6**, supporting (i) impaired cell viability and (ii) reduced tumor mass. The same results were obtained when glucose uptake was analyzed *ex vivo* on explanted tumors (**Supplementary Figures 2A, B**).

**Figure 7:**
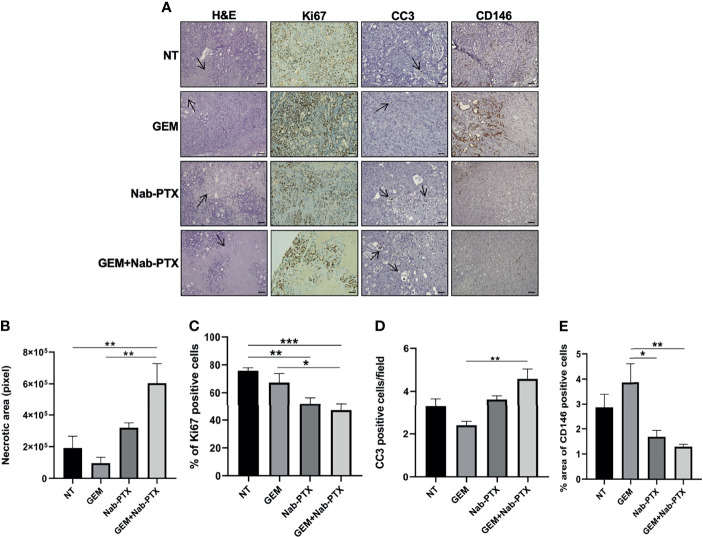
Effect of GEM + Nab-PTX on necrosis, proliferation, apoptosis, and tumor vascularization. **(A)** Representative images of H&E (magnification ×10) staining and IHC (Ki67, CC3, and CD146, magnification ×20). Scale bar, 100 µM. Treatments: NT (vehicle), GEM (25 mg/kg), Nab-PTX (10 mg/kg), GEM (25 mg/kg) + Nab-PTX (10 mg/kg). **(B)** Necrotic areas expressed in pixel. **(C)** Percentage of Ki67-positive cells. **(D)** Number of CC3-positive cells per field. **(E)** Percent area of CD146-positive cells. All the histograms represent the mean ± SEM of the values *p < 0.05; **p < 0.01; ***p < 0.001.

To dissect the antitumor activity of GEM and Nab-PTX association, we characterized tumor morphology in the different treatment arms. H&E staining of xenograft sections (**Figure 7**) showed an increase in necrotic areas in GEM + Nab-PTX-treated tumors compared to both NT (p < 0.01) and GEM (p < 0.01). IHC analysis revealed a lower proliferation rate (**Figures 7A, C**), as quantified by Ki67 staining, in the drug combination arm in comparison with both NT (p < 0.001) and GEM (p < 0.05) and in the Nab-PTX monotherapy arm compared to NT (p < 0.01). The results of CC3 staining (**Figures 7A, D**) were consistent with those obtained *in vitro*, with an increase of apoptosis in the combination arm with respect to GEM (p < 0.01). Lastly, we evaluated tumor vasculature by staining for the endothelial cell marker CD146 (**Figures 7A, E**); results indicated a significant role of both Nab-PTX (p < 0.05) and Nab-PTX + GEM (p < 0.01) in impairing tumor blood vessels compared to GEM.

Together, these results demonstrate the efficacy of a Nab-PTX-including drug combination in reducing tumor growth in a xenograft model of multidrug-resistant iCCA in which GEM or PTX monotherapy is ineffective.

## Discussion

The onset of chemoresistance is the main cause of failure in the management of CCA. To ensure an effective second-line treatment, the identification of alternative therapeutic options to overcome resistance and/or to restore sensitivity to standard chemotherapy is mandatory (4, 5).

In our recent studies, we have isolated and characterized two GEM-resistant cell lines derived from iCCA patient-derived xenografts. These cells, named MT-CHC01R1.5 and 82.3, are refractory to 5-FU (13, 14)—a drug commonly used in second-line therapeutic regimens—as well as to carboplatin and trabectedin. These features make them *bona fide* multidrug-resistant preclinical models of iCCA. Starting from a drug prediction analysis on MT-CHC01R1.5 cells, we have identified PTX as a potentially effective treatment alternative (14). In the present work, we demonstrate the preclinical antitumor activity of PTX, as monotherapy and/or in combination with GEM.

*In vitro*, we observed a potent anti-proliferative effect of PTX on our multidrug-resistant iCCA cell lines, with IC50 values in line with those described in the literature for other CCA models and other cancer types (31). Accordingly, in both iCCA cell lines, PTX overcomes GEM resistance by (i) affecting the cell cycle, (ii) inducing apoptosis mediated by CC3 and cPARP, and (iii) reducing the stem cell compartment. The behavior of each cell line was peculiar, in terms of both response to a specific drug regimen and mechanism of action, which requires further discussion.

PTX is known to stabilize cancer cells in the mitotic phase by blocking them in G2/M, as observed in most preclinical tumor models (16, 32). In 82.3 cells, the response to single-agent PTX was in line with this observation. In MT-CHC01R1.5 cells, instead, this same treatment caused a mild decrease in the G0/G1 phase and a proportional increase in the S phase. Although not common, a block in the S phase has already been observed in pancreatic tumor cell lines resistant to GEM after PTX exposure (33). In our opinion, this outcome may indicate a marginal cytostatic effect due to the poor efficacy of single-agent PTX in this cell model. Treatment with GEM + PTX provoked a prominent block in G2/M in MT-CHC01R1.5 cells, while—unexpectedly—it had no effect on 82.3 cells. Similarly, single-agent PTX induced apoptosis in both cell lines while the combination, again, was effective on MT-CHC01R1.5 cells but not on 82.3 cells. These results highlight the relevance of drug interaction: while GEM + PTX combination resulted in cytostatic and cytotoxic effects in MT-CHC01R1.5 cells, the presence of GEM antagonized PTX in 82.3 cells, nullifying its efficacy.

The fact that effective cell cycle arrest and apoptosis are reachable in models of multidrug-resistant iCCA by applying different regimes implies that divergent molecular and cellular mechanisms may be involved. These mechanisms are far from being understood. In our previous work, we reported the upregulation of *TUBB2* in iCCA-resistant models (14); this gene codifies for the β2 chain of tubulin (15, 16). Likewise, Le Large and collaborators found elevated levels of microtubule-associated protein 2 (MAP2) in GEM-resistant pancreatic cancer models that were highly sensitive to PTX both *in vitro* and *in vivo* (34). Our data strengthen the concept that drug resistance is a multivariate phenomenon, in which a similar outcome may result from alternative molecular mechanisms. In the cholangiosphere formation assay, PTX reduced the number of spheres in both cell lines, implying an impact on the stem cell compartment as a further mechanism of drug re-sensibilization. In 83.2, the reduction of cholangiosphere formation was also observed upon GEM treatment, probably due to the presence of a subgroup of drug-sensitive cells in this highly heterogenic cell line (13). Unfortunately, the scarcity of multidrug-resistant iCCA preclinical models does not allow comparative molecular studies to identify the most relevant mechanisms of resistance and/or a possible implication of primary *versus* acquired refractoriness to GEM. So, at least for now, the molecular studies remain incomplete and somehow puzzling. To try to overcome this limit, we are in the process of characterizing a small panel of other cell lines isolated from patients in our Institute and stabilized in our laboratory.

For the *in vivo* studies, we treated MT-CHC01R1.5-xenografted mice with Nab-PTX, an albumin-based nanocarrier formulation. Despite the similar efficacy of PTX and Nab-PTX *in vitro* (30), a series of preclinical and clinical studies demonstrated that the latter is more beneficial than PTX *in vivo*, due to (i) more efficient transendothelial transport, (ii) higher bioavailability and lower toxicity in xenograft models, and (iii) increased extravascular distribution in patients (35). In addition, a combination of Nab-PTX and GEM is currently being tested in clinical trials as first-line treatment for patients with advanced/metastatic CCA (20, 21). Our *in vivo* experiments mirrored what was observed *in vitro* on cultured MT-CHC01R1.5 cells. Single-agent Nab-PTX was unable to delay tumor growth in terms of either volume or weight and had only a marginal effect on tumor vasculature. In contrast, the simultaneous administration of GEM and Nab-PTX drastically slowed the growth curve, resulting in tumors with larger necrotic areas, reduced numbers of proliferating cells, increased numbers of apoptotic cells, and decreased numbers of vascular endothelial cells. The outcome of glucose uptake evaluation, an assay similar to ^18^F-fluoro-2-deoxy-D-glucose positron emission tomography ([18]FDG-PET) scanning commonly used in patients for tumor follow-up, confirmed the efficacy of the combination regimen.

A limit of our *in vivo* studies is the lack of other tumorigenic GEM-resistant iCCA models, which is a reflection of the rarity of this tumor. The other cell line tested *in vitro*, 82.3, is not amenable for *in vivo* investigation due to its long engraftment latency in NOD/SCID mice and poor reproducibility of tumor growth (13). Another resistant iCCA cell line available in our laboratory, HuH28 (36, 37), is not tumorigenic *in vivo*. Finally, the majority of cell lines that might be accessed through collaborations have been isolated from Asian patients, who are expected to have different genetic and dietary characteristics from our Italian cohort, therefore not being suitable for comparative studies. The development of new and appropriate models of multidrug resistance will be crucial to evaluating possible therapeutic alternatives in refractory iCCAs.

## Conclusions

Collectively, the results presented here support the use of PTX in multidrug-resistant iCCA settings. This approach is not expected to represent a life-saving strategy but may improve disease control in stratified cohorts of patients. However, because the optimal *in vitro* efficacy of PTX was achieved with different drug regimens—combination on MT-CHC01R1.5 cells and single agent on 82.3 cells—a careful evaluation of multidrug regimens should precede the patient stratification in forthcoming pilot studies. To this aim, a broader characterization of the mechanisms, in terms of signaling pathways affected and/or residual sensitivity to other drugs and drug combinations, is necessary and will be the subject of future studies in our laboratory.

## Data Availability Statement

The original contributions presented in the study are included in the article/**Supplementary Material**. Further inquiries can be directed to the corresponding author.

## Ethics Statement

The animal study was reviewed and approved by 177/2015-PR, 178/2015-PR, and 106/2021-PR.

## Author Contributions

AM, CPN, FV, and GC performed the experimental designs,many experiments, and data acquisition/interpretation and drafted the manuscript. AM, CPN, FV, and CV performed various in vitro experiments. MB performed the in vivoexperiments. GC, FL, SM and MA critically contributed to the research unwinding. PB performed the cytofluorimetric analysis. JE and IS performed the imaging and histologic analysis. CR supplied the tumor specimens and critically edited the manuscript. FL, GC, CPN, MA and SM critically edited the manuscript. GC and MA conducted the scientific leadership. All authors contributed to the article and approved the submitted version.

## Funding

AM was supported by the “Roche per la Ricerca” grant funded by the Roche Foundation and by a “Nino Consigliere” fellowship funded by Fondazione Piemontese per la Ricerca sul Cancro Onlus (FPRC). CPN was supported by “Compagnia di San Paolo”. This work was supported by FPRC 5xmille 2015 Ministero Salute “Cancer Im-Gen” grant and “Ricerca Corrente 2020 Ministero della Salute” (to MA) and by “Associazione Italiana per la Ricerca sul Cancro-Investigator Grant” (AIRC-IG 2018 number 21679) (to SM).

## Conflict of Interest

The authors declare that the research was conducted in the absence of any commercial or financial relationships that could be construed as a potential conflict of interest.

## Publisher’s Note

All claims expressed in this article are solely those of the authors and do not necessarily represent those of their affiliated organizations, or those of the publisher, the editors and the reviewers. Any product that may be evaluated in this article, or claim that may be made by its manufacturer, is not guaranteed or endorsed by the publisher.

## Abbreviations

CCA, cholangiocarcinoma; GEM, gemcitabine; PTX, paclitaxel; 5-FU, 5-fluorouracil; iCCA, intrahepatic cholangiocarcinoma; pCCA, perihilar cholangiocarcinoma; dCCA, distal cholangiocarcinoma; HCC, hepatocellular carcinoma; EMT, epithelial–mesenchymal transition; FP, fluoropyrimidine; ORR, overall response rate; PFS, progression-free survival; aCCA, advanced cholangiocarcinoma; NAB, albumin-bound nanoparticle; OS, overall survival.
