# Supplementary material for: Paclitaxel Restores Sensitivity to Chemotherapy in Preclinical Models of Multidrug-Resistant Intrahepatic Cholangiocarcinoma
Source: Front Oncol. 2022 Feb 17;12:771418. doi: 10.3389/fonc.2022.771418 (PMC8891641; doi:10.3389/fonc.2022.771418)
Supplement: Supplementary file 1 [file DataSheet_1.docx]

**SUPPLEMENTARY FIGURE 1**

**
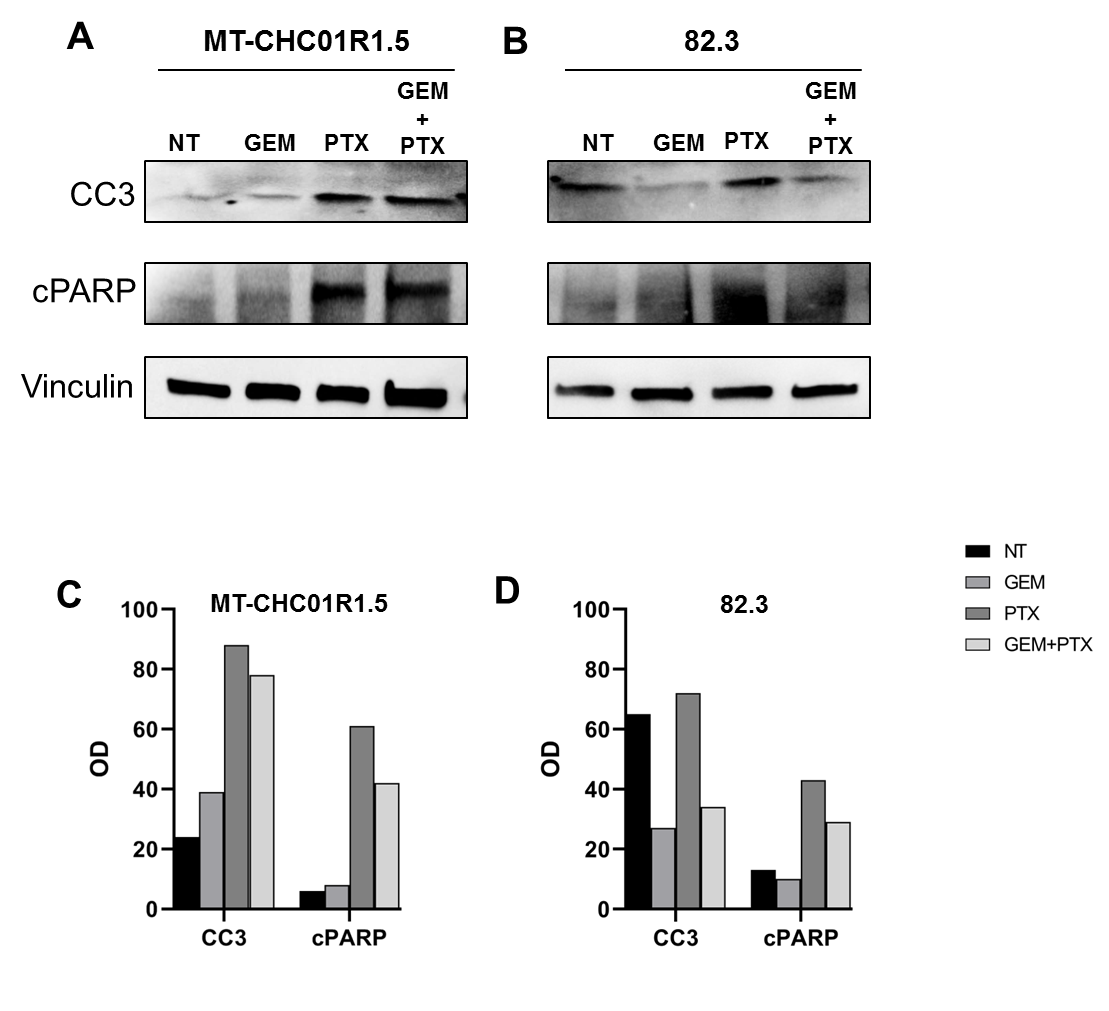
**

**Supplementary Figure 1.** Western blot analysis for the expression of apoptotic markers, CC3 and PARP, on MT-CHC01R1.5 (A) and 82.3 (B) cell lines, untreated and treated with GEM (1.5 µM), PTX (15 ng/mL), or GEM + PTX. Densitometric quantification of western blot band intensity of MT-CHC01R1.5 (C) and 82.3 (D) cells.

**SUPPLEMENTARY FIGURE 2**

**
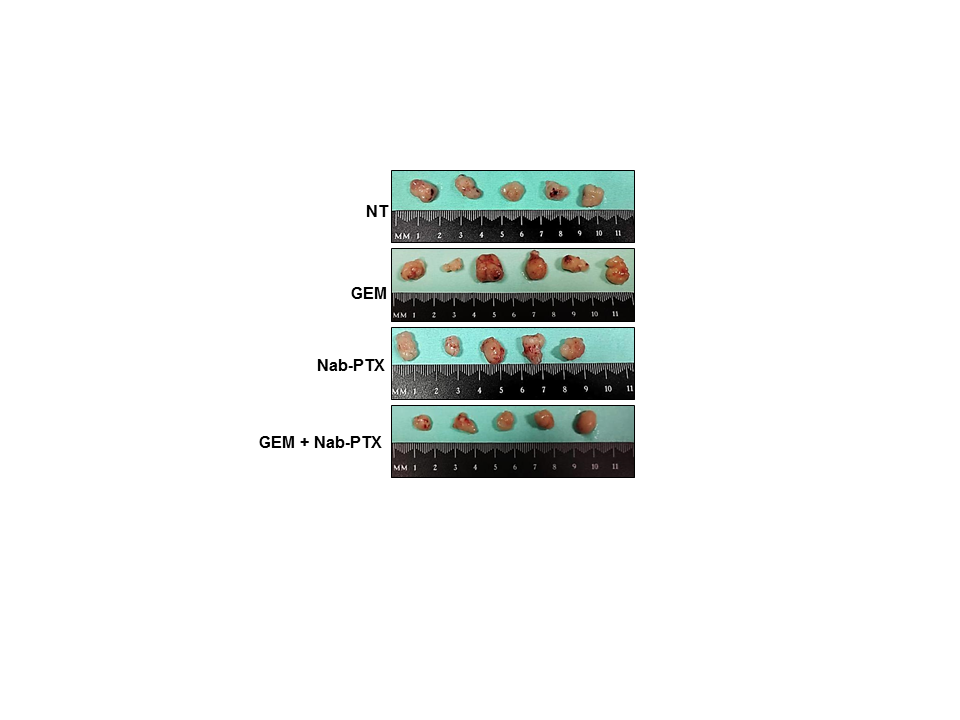
**

**Supplementary Figure 2.** Representative explanted tumors after sacrifice (NT: treated with vehicle; GEM: Gemcitabine 25 mg/kg; Nab-PTX 10 mg/kg; GEM 25 mg/kg + Nab-PTX 10 mg/kg).

**SUPPLEMENTARY FIGURE 3**


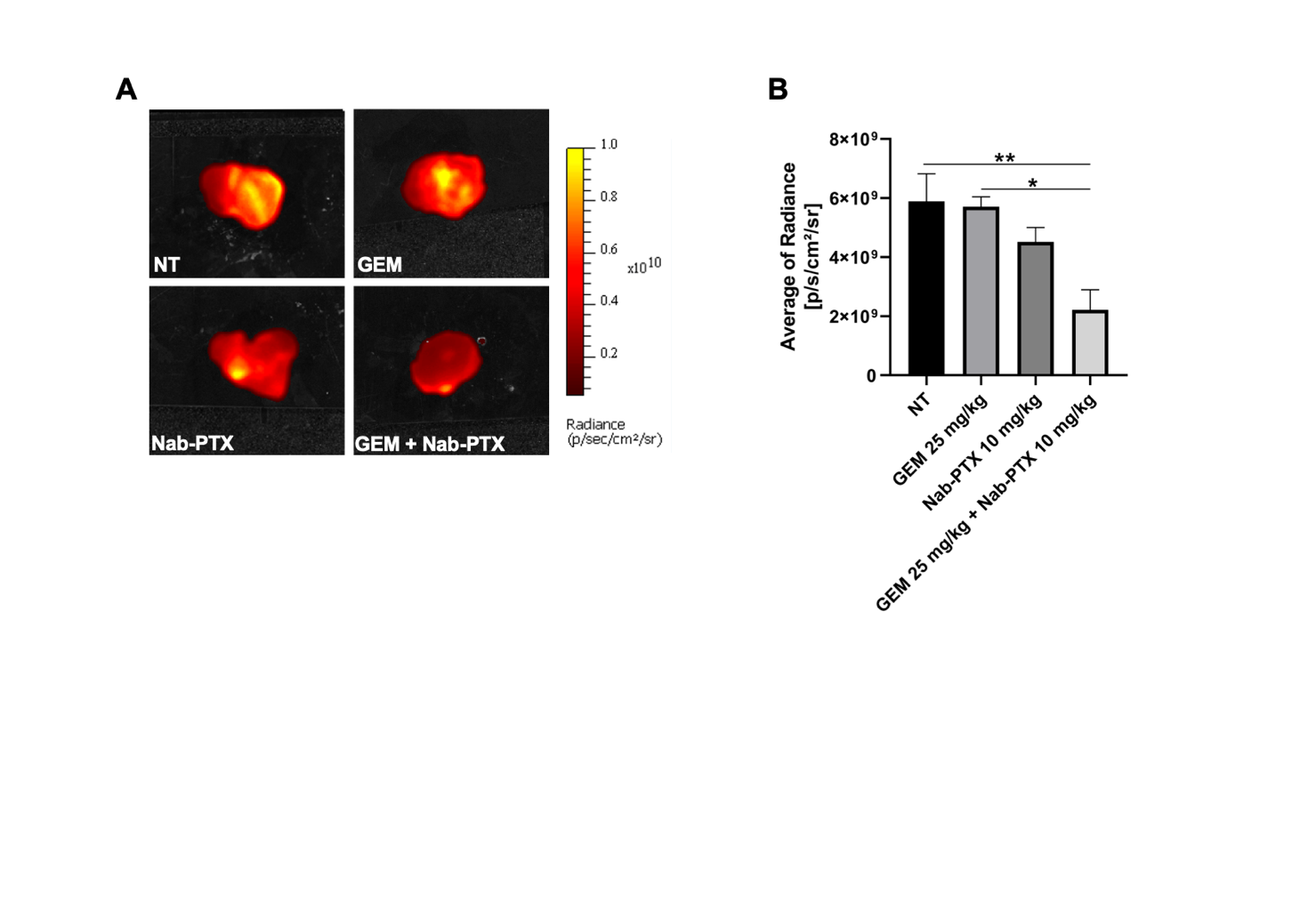


**Supplementary Figure 3. GEM and Nab-PTX combination effect on *ex vivo* glucose uptake. A)** Representative images of *ex vivo* tumor-glucose uptake (NT; GEM 25 mg/kg; Nab-PTX 10 mg/kg; GEM 25 mg/kg + Nab-PTX 10 mg/kg). **B)** Histograms reporting average of radiance expressed in p/s/cm^2^/sr of the indicated treatments. One-way Anova was used to calculate statistical significance (∗ p<0.05, ∗∗ p<0.01).

Supplementary Table 1. Cell cycle analysis in MT-CHC01R1.5 and 82.3 cell lines.

| **MT-CHC01R1.5** | | | | **82.3** | | | |
| --- | --- | --- | --- | --- | --- | --- | --- |
| **Mean % ± SD p-value** | | | | **Mean % ± SD p-value** | | | |
| **NT** | G0/G1 | 81.99 ± 2.50 |  | **NT** | G0/G1 | 46.39 ± 1.49 |  |
|  | S | 6.85 ± 1.13 |  |  | S | 22.40 ± 5.76 |  |
|  | G2/M | 8.62 ± 1.13 |  |  | G2/M | 28.88 ± 4.79 |  |
| **GEM** | G0/G1 | 78.74 ± 1.96 |  | **GEM** | G0/G1 | 63.03 ± 1.68 | GEM vs NT ** |
|  | S | 8.21 ± 1.04 |  |  | S | 10.85 ± 6.33 |  |
|  | G2/M | 10.02 ± 2,22 |  |  | G2/M | 21.52 ± 6.19 |  |
| **PTX** | G0/G1 | 58.56 ± 3.51 | PTX vs NT vs GEM * | **PTX** | G0/G1 | 14.38 ± 14.12 | PTX vs GEM * |
|  | S | 18.16 ± 2.05 | PTX vs NT vs GEM * |  | S | 33.75 ± 2.72 | PTX vs GEM * |
|  | G2/M | 9.88 ± 0.74 | NS |  | G2/M | 39.48 ± 18.17 | NS |

One-way Anova test was used for statistical analysis (∗ p<0.05; ∗∗ p< 0.01).
